# PGE2 Enhances Human ILC3 Function but Constrains ILC2‐to‐ILC3 Plasticity

**DOI:** 10.1002/eji.70296

**Published:** 2026-10-08

**Authors:** Lorenz Wirth, Whitney Weigel, Efthymia Kokkinou, Johan Kolmert, Anna‐Karin Johnsson, Alessandro Quaranta, Craig Wheelock, Mattias Jangard, Ram V. Pandey, Sven‐Erik Dahlén, Christopher Andrew Tibbitt, Thomas Hochdörfer, Jenny Mjösberg

**Affiliations:** ^1^ Center for Infectious Medicine Department of Medicine Huddinge Karolinska Institutet and Karolinska University Hospital Stockholm Sweden; ^2^ Mechanistic Biology & Profiling Discovery Sciences R&D AstraZeneca Gothenburg Sweden; ^3^ Clinical Lung‐ and Allergy Research Unit Department of Medicine Huddinge Karolinska Institutet Stockholm Sweden; ^4^ Department of Oncology Bloomberg Kimmel Institute of Cancer Immunotherapy Johns Hopkins University School of Medicine Baltimore Maryland USA; ^5^ Unit of Integrative Metabolomics Institute of Environmental Medicine Karolinska Institutet Stockholm Sweden; ^6^ Division of Immunology and Allergy Department of Medicine Solna and Center for Molecular Medicine Karolinska Institutet and Karolinska University Hospital Stockholm Sweden; ^7^ Department of Respiratory Medicine and Allergy Karolinska University Hospital Stockholm Sweden; ^8^ ENT Unit Sophiahemmet University and Sophiahemmet Hospital Stockholm Sweden; ^9^ Department of Dermatology Medical University of Vienna Vienna Austria

**Keywords:** ILC2, ILC3, PGE2, plasticity, prostaglandins

## Abstract

Type 2 innate lymphoid cells (ILC2) drive eosinophilic asthma, whereas ILC3 and plastic ILC2/3 states are implicated in neutrophilic and mixed granulocytic disease endotypes. While lipid mediators regulate ILC2 function and airway inflammation, their role in human ILC3 biology and ILC plasticity remains poorly defined. We investigated how eicosanoid biosynthesis and signalling regulate human ILC2 and ILC3 function across canonical and plastic states. Here, we identified distinct prostaglandin (PG) profiles across ILC subsets. Unlike ILC2, which produce and rely on PGD_2_, ILC3 produced PGE_2_, which stimulated their release of IL‐17F and IL‐22. ILC2‐to‐ILC3 plasticity induced a biosynthetic switch towards PGE_2_ production that correlated with IL‐17A and IL‐17F expression. However, subset‐intrinsic prostaglandin responses were preserved: hybrid ILC2/3 phenotypes, unlike *bona fide* ILC3, remained susceptible to PGE_2_‐mediated inhibition, indicating a key functional distinction between these populations.Collectively, our data identify PGE_2_ as a central regulator of ILC3 function, paralleling the role of PGD_2_ in ILC2. These findings position PGs as key orchestrators of ILC subset function and plasticity and provide mechanistic insights into the regulation of ILC3 and hybrid ILC2/3 phenotypes associated with neutrophilic and mixed granulocytic asthma.

## Graphical abstract

Distinct prostaglandin circuits regulate human ILC2 and ILC3 function. ILC3‐derived PGE_2_ promotes type 3 cytokine production, whereas hybrid ILC2/3 generated during plasticity retain an inhibitory response to PGE_2_ despite acquiring ILC3‐like features.

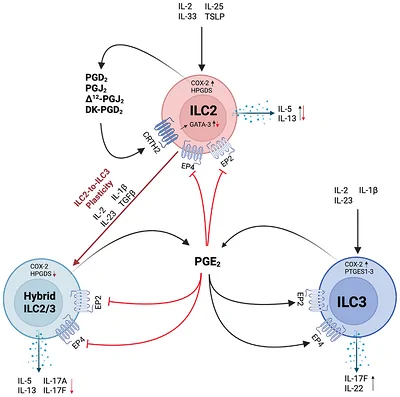

**Abbreviations**
FACS: fluorescence‐activated cell sorting
FI: fluorescence intensity
GEO: Gene Expression Omnibus
ILC(s): innate lymphoid cell(s)
ILC2: group 2 ILCs
ILC3: group 3 ILCs
LC‐MS/MS: liquid chromatography‐tandem mass spectrometry
LTD_4_
: leukotriene D_4_
LTi: lymphoid tissue inducer (cells)
NHS: normal human serum
NK: NK (cells)
P/I: PMA/ionomycin
P/S: penicillin/streptomycin
PBMC(s): peripheral blood mononuclear cell(s)
PG(s): prostaglandin(s)
PGD_2_
: prostaglandin D_2_
PGE_2_
: prostaglandin E_2_
PUFA(s): polyunsaturated fatty acid(s)

## 1 Introduction

Innate lymphoid cells (ILCs) constitute a rare population of immune cells and can be broadly grouped into natural killer (NK) cells, type 1–3 ILCs (ILC1‐3), and lymphoid tissue inducer (LTi) cells [1]. Lacking specific antigen receptors, ILC function is driven by cues derived from their microenvironment [1]. Extensive studies have characterized the diverse signals regulating ILC function, which prominently include cytokines released by epithelial cells and other innate immune cells [1, 2, 3]. For ILC2, these prominently include the alarmins IL‐33, IL‐25, and TSLP [4, 5], while ILC3 have been shown to respond to IL‐1β and IL‐23 [6, 7].

In addition to cytokines, eicosanoids, bioactive lipids derived from arachidonic acid and related polyunsaturated fatty acids (PUFAs), have also emerged as potent regulators of ILC2 function, promoting [8, 9, 10] or suppressing [11] their activity. Further highlighting the significance of eicosanoids for ILC2 biology, we previously showed that ILC2s produce prostaglandin D_2_ (PGD_2_) upon alarmin stimulation, driving an auto‐/paracrine loop that is essential for their activation [12]. While eicosanoids have been well established as central drivers and regulators of ILC2 function [13], their role for other ILC subsets remains poorly understood. Recent studies demonstrated that prostaglandin E_2_ (PGE_2_) supports ILC3 function in mice. Signalling via the PGE_2_ receptor EP4 was shown to induce IL‐22 in ILC3 [14], while autocrine PGE_2_ mediated protective functions of ILC3 in the intestine [15]. However, it remains unclear whether human ILC3 produce or respond to PGE_2_, and how these pathways compare with those operating in ILC2.

Aberrant eicosanoid signalling has been implicated in chronic inflammatory diseases. Type 2‐associated inflammatory eicosanoids such as PGD_2_ and leukotriene D_4_ (LTD_4_) contribute to exacerbation and disease severity in patients with asthma [16, 17, 18], while PGE_2_ was found elevated in the intestines of individuals with Crohn's disease [19] and in psoriatic skin [20]. Similarly, dysregulated ILC responses have been linked to chronic inflammatory conditions. As such, ILC2 support airway inflammation in type 2‐high asthma via production of IL‐5 and IL‐13 [16, 17, 21, 22, 23], while ILC3 composition is altered in the intestine of patients with inflammatory bowel disease, skewing towards reduced NKp44^+^ ILC3 [24, 25, 26, 27, 28].

ILCs display a considerable potential for plasticity, allowing adaptation to their local microenvironment [29] and the acquisition of features associated with other ILC subsets [30]. Plasticity between ILC2 and ILC3 has been suggested in inflammatory conditions [31, 32, 33]. In mixed granulocytic asthma, a hybrid ILC2‐ILC3 phenotype was recently described, co‐expressing hallmark type 2 and type 3 cytokines IL‐5 and IL‐13 as well as IL‐17A and correlating with neutrophil numbers in sputum [33].

In this study, we dissected endogenous eicosanoid synthesis and function across human ILC subsets. We demonstrate subset‐specific prostaglandin production, with ILC2 producing PGD_2_, while ILC3 release PGE_2_ upon cytokine stimulation, which engages an auto‐/paracrine loop that enhances their expression of IL‐17F and IL‐22. Notably, PGE_2_ synthesis was part of the type 3 program induced during ILC2‐to‐ILC3 plasticity. However, the inhibitory effect of PGE_2_ on ILC2 was conserved, revealing incomplete functional reprogramming. Together, our findings reveal that eicosanoids have a broader regulatory function in ILC biology than previously recognized and offer new insights into the features and limitations of ILC plasticity. A deeper understanding of how eicosanoid signaling and metabolism shape ILC function and fate may inform new therapeutic strategies for inflammatory conditions characterized by dysregulated ILC responses.

## 2 Methods

### 2.1 Analysis of RNA‐Sequencing Data

Publicly available bulk RNA‐sequencing data on ILC subsets were obtained from the NCBI Gene Expression Omnibus (GEO; accession number GSE124474) as a transcripts per million (TPM) count matrix, mapped to CRCh38.p13. Heatmaps were generated using the pheatmap package in R.

### 2.2 Analysis of Affymetrix Microarray Data

Publicly available Affymetrix Clariom S Assay HT microarray data (GEO; accession number GSE12923) on human blood‐derived ILC2 stimulated with ILC3‐polarizing cytokines were imported into R using the oligo package [34]. Data were normalized via the Robust multi‐array average algorithm [35], performing background correction, quantile normalization, and probe‐level summarization. Probe set annotation was conducted using the *clariomdhumantranscriptcluster.db* package (MacDonald, 2021). Probe sets without Entrez Gene identifiers were excluded, and redundant mappings were collapsed by averaging expression values for identical Entrez IDs with the *affycoretools::avereps* function [36]. Differential expression analysis was performed using the limma package, fitting a linear model to the log_2_‐transformed normalized expression values, and performing a moderated *t*‐test with an empirical Bayes trend adjustment. *p*‐values were adjusted for multiple testing using the Benjamini‐Hochberg False Discovery Rate method. Volcano plots were generated using ggplot2 and ggrepel.

### 2.3 Isolation of Human ILC2 and ILC3 From Tonsil and Blood

Human ILC2 were isolated from peripheral blood or tonsils; ILC3 were isolated from human tonsils. Blood was sourced from healthy donors at the Karolinska University Hospital blood bank. Tonsils were obtained from patients undergoing resection surgery for obstructive sleep apnea. All work was approved by the Swedish Ethical Review Authority. Briefly, peripheral blood mononuclear cells (PBMCs) were isolated via Ficoll density gradient centrifugation. CD3^+^ cells were depleted using CD3 MACS microbeads and LD‐columns, followed by positive selection for CD127^+^ cells using CD127 MACS microbeads and LS‐columns (all Miltenyi Biotec). Tonsils were mechanically dissociated and filtered through 100 µm cell strainers using the blunt end of a syringe. Mononuclear cells were isolated using Ficoll density gradient centrifugation. CD3^+^, CD14^+^, and CD19^+^ cells were subsequently depleted using MACS microbeads and LD‐columns (all Miltenyi Biotec). Following surface staining (Table S1), ILC2 and ILC3 were isolated by fluorescence‐activated cell sorting (FACS) using a Sony MA900 cell sorter (Figure S1).

### 2.4 In Vitro Culture and Stimulation of ILC3

FACS‐sorted ILC3 were cultured in Yssel's‐supplemented IMDM (2% normal human serum (NHS), 1% penicillin‐streptomycin (P/S)) and stimulated with IL‐2 (10 ng/mL; Peprotech), IL‐1β (R&D Systems), and IL‐23 (Peprotech) (both 50 ng/mL), alone or in combination. PGE_2_ (100 nM; Sigma‐Aldrich) was spiked into the indicated cultures, and/or the EP2 antagonist PF‐04418948 and the EP4 antagonist ONO‐AE3‐208 (both 1 µM; Cayman Chemical), with DMSO serving as a vehicle control.

### 2.5 In Vitro Expansion of ILC2 and ILC3

Sorted cells were expanded as previously described [37]. Briefly, 1000 cells were seeded in 200 µL of expansion medium (Yssel's supplemented IMDM, 2% NHS, 1% P/S) supplemented with IL‐2 (100 ng/mL), IL‐7 (10 ng/mL) (both PeproTech), and IL‐1β (50 ng/mL; R&D Systems) in 96‐well U‐bottom plates. Following a half‐medium exchange (on days 5–6) and splitting (days 10–11), the cells were harvested at day 14. Expanded cells were counted and reseeded at 250,000 cells per 96 U‐well in 200 µL of resting medium (Yssel's supplemented IMDM, 2% NHS, 1% P/S) supplemented with IL‐2 (2 ng/mL) and IL‐7 (5 ng/mL). After 1–2 days of resting, the cells were collected from the wells, counted, and used for subsequent experiments.

### 2.6 Mass Spectrometric Analysis of Culture Supernatants and Intracellular Lipid Extracts

For liquid chromatography‐tandem mass spectrometry (LC‐MS/MS) assays, cells were cultured in IMDM (2% NHS, 1% P/S) without Yssel's supplementation to minimize background interference. Oxylipins were extracted from supernatants (200 µL) by adding 10 µL deuterated internal standard mix followed by dilution with 800 µL extraction buffer. Intracellular lipids were extracted by quenching the cells with 200 µL ice‐cold methanol. Following incubation on ice for 20 min, the samples were centrifuged at 10,600 × *g* for 10 min, and supernatants were collected. For analysis, the supernatants were evaporated to dryness with nitrogen gas and redissolved in 1 mL extraction buffer and 10 µL internal standard mix. Solid‐phase extraction was performed using an Evolute Express ABN, 60 mg, 3 mL cartridge operated by the Extrahera automated system (Biotage). Eluates were dried under nitrogen, redissolved in methanol:water (6:1, v/v), and filtered using spin filters (Amicron, Merck Millipore). Oxylipin profiling was performed by targeted quantification using an Acquity UPLC coupled to an Xevo‐TQ‐XS mass spectrometer operated in negative ion electrospray mode. An external calibration curve mix of eleven levels was used to ensure a minimum of six calibration points for each reported analyte. Further details of the analytical method are provided in the supplementary information. Cell numbers for LC‐MS/MS analysis varied between donors (1.5–2.5 × 10^5^ cells per donor) due to differences in expansion efficiency. Within each donor, equal cell numbers were used across conditions, and measured lipid concentrations were normalized to 2.5 × 10^5^ cells.

### 2.7 ILC2‐To‐ILC3 Plasticity Assay

For plasticity cultures, ILC2 were seeded at 1000 cells per well in 50 µL of Yssel's‐supplemented IMDM (2% NHS, 1% P/S) in 96‐well U‐bottom culture plates. Control conditions were either stimulated with IL‐2 (10 ng/mL) and IL‐7 (5 ng/mL) (both PeproTech) alone (“Ctrl”), or additionally with 50 ng/mL of each of IL‐1β, IL‐25 (both R&D Systems), IL‐33, and TSLP (PeproTech) (“Pro2”). For ILC3 polarization (“Pro3”), cells were stimulated with IL‐2 (10 ng/mL), IL‐7 (5 ng/mL), and IL‐1β, IL‐23, and TGFβ (each 50 ng/mL; all R&D Systems). PGE_2_ (100 nM; Sigma‐Aldrich), EP2 antagonist PF‐04418948, and EP4 antagonist ONO‐AE3‐208 (both 1 µM; Cayman Chemical) were spiked into the indicated conditions. Cultures were restimulated on day 3 by spiking in the corresponding cytokines and compounds. On day 6, the medium was exchanged, and cells were restimulated for 24 h. For the final 3 h, PMA (Sigma‐Aldrich), ionomycin (Invitrogen) (P/I), and Golgi‐Plug and Golgi‐Stop (both BD Biosciences) were added. Subsequently, supernatants were collected and snap‐frozen on dry ice, and cells were stained for flow‐cytometric analysis.

### 2.8 CellTrace Proliferation Assay

To quantify proliferation of plasticity cultures, the cells were stained with CellTrace Violet stain (5 µM; Invitrogen) for 25 min at 37°C on day 0, according to the manufacturer's protocol. CellTrace dilution was subsequently assessed by flow cytometry after completion of the plasticity cultures.

### 2.9 ELISA

IL‐17F and IL‐22 levels were assessed using DuoSet ELISA kits together with the DuoSet Ancillary Reagent Kit 2 (all from R&D Systems). PGE_2_ and PGD_2_ levels were analyzed using Prostaglandin D_2_ and E_2_ ELISA kits (Cayman Chemical).

### 2.10 Flow Cytometry Analysis

Surface marker staining was performed for 30 min at RT (Table S2). Subsequently, cells were fixed in 2% PFA (Thermo Scientific) for 7 min at RT. Intracellular staining was performed using BD FACS permeabilizing solution 2 or the BD Cytofix/Cytoperm fixation/permeabilization kit (both BD Biosciences). Intracellular staining was performed for 30 min at RT. For cytokine analysis of ILC3 ex vivo stimulations, a median of 2224 total live cells, and for cytokine analysis of ILC2‐to‐ILC3 plasticity, a median of 1284 total live cells were subsequently acquired during flow cytometry. For staining transcription factors, cells were fixed and permeabilized following surface marker staining using the eBioscience Foxp3 Transcription Factor Fixation/Permeabilization kit. Transcription factor staining was performed overnight at 4°C. Samples were analyzed on a BD FACSymphony A5 or LSR II Fortessa cell analyzer, running FACSDiva Software. Data analysis was performed with FlowJo v10.10.0 (BD).

## 3 Results

### 3.1 Human ILC3 Produce PGE_2_

Given the indispensable role of prostaglandin biosynthesis and signalling for human ILC2 function, we investigated whether other ILC subsets possess analogous oxylipin‐dependent regulatory circuits. Reanalysis of human RNA‐sequencing data revealed that both NKp44^+^ and NKp44^−^ ILC3 subsets expressed key components required for PGE_2_ biosynthesis, including *PLA2G4A*, *PTGS2* (encoding COX‐2), and the PGE_2_ synthases *PTGES2* and *PTGES3* (Figure 1A,B; Figure S2). In addition, ILC3 expressed genes associated with prostaglandin transport, such as *ABCC4* and *SLCO2A1* (Figure 1B). Similar to ILC2, which upregulate COX‐2 upon activation [12], resting ILC3 lacked COX‐2 protein expression (Figure 1C). However, IL‐2, IL‐1β, and IL‐23 synergistically induced COX‐2 expression (Figure 1D,E), indicating that inflammatory cues promote activation of the prostaglandin biosynthetic pathway in ILC3.

**FIGURE 1:**
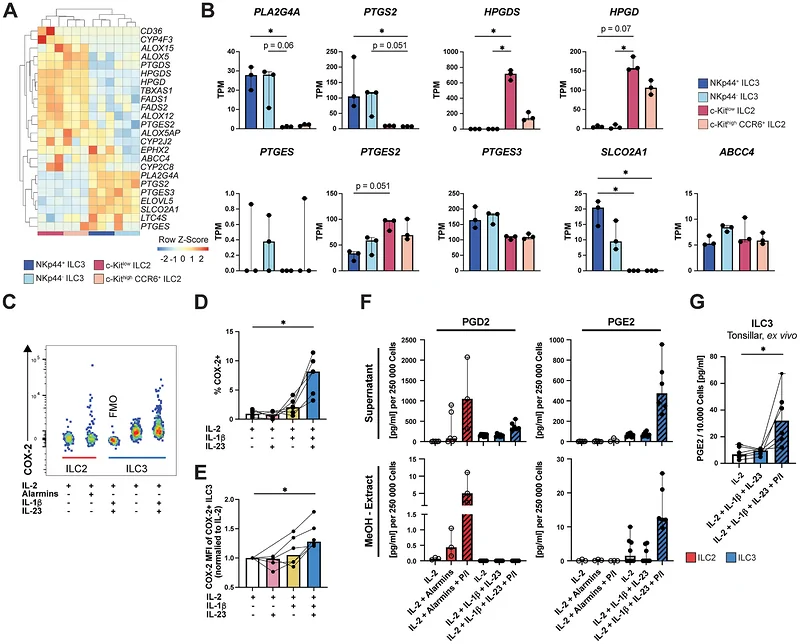
Human ILC3 produce PGE_2_. (A) Heatmap of genes involved in eicosanoid synthesis and transport in tonsillar NKp44^+/−^ ILC3 and blood‐derived c‐Kit^low/high^ ILC2 (*n* = 3 per subset). Color scale represents row Z‐scores of TPM values. (B) Expression of selected genes involved in prostaglandin synthesis, degradation, and transport in ILC3 and ILC2. Data are expressed as transcripts per million (TPM); statistical significance was determined using Kruskal–Wallis with Dunn's multiple comparisons test. (C) Representative flow cytometric plots of COX‐2 expression in tonsillar ILC2 and ILC3 after 24 h ex vivo stimulation. (D) Frequency of COX‐2^+^ ILC3 and (E) COX‐2 MFI in COX‐2^+^ ILC3 after 24 h ex vivo stimulation with cytokine combinations (*n* = 5–6). MFI values were normalized to the IL‐2 control. COX‐2 frequencies were compared using the Friedman test with multiple comparisons. COX‐2 MFIs of IL‐2 control and full cytokine mix were compared using the Wilcoxon matched‐pairs signed‐rank test. (F) PGD_2_ and PGE_2_ concentrations in ILC2 (*n* = 3) and ILC3 (*n* = 8 and *n* = 6 for P/I‐stimulated conditions) culture supernatants and cell extracts from expanded tonsillar ILC subsets, stimulated for 24 h with subset‐specific stimuli, as measured by mass spectrometry. (G) PGE_2_ levels in tonsillar ILC3 stimulated ex vivo for 24 h (*n* = 6). For ELISA measurements, 25,000–50,000 ILC3 were used per condition. PGE_2_ concentrations were normalized to 10,000 cells. Statistical differences between the groups were tested using the Friedman test with multiple comparisons. All bars show median and range. Significance levels: *p < 0.05.

To determine whether activated ILC3 produce prostaglandins, we performed LC‐MS/MS analysis on supernatants and cell extracts from expanded ILC cultures (Figure S3). In contrast to alarmin‐stimulated ILC2, which predominantly produced PGD_2_ (Figure 1F), alongside smaller quantities of PGD_1_, PGJ_2_, Δ^12^‐PGJ_2_, and thromboxane B_2_ (Figure S3B), ILC3 stimulated with IL‐2, IL‐1β, IL‐23, and PMA/ionomycin produced PGE_2_, which was detected in both supernatants and intracellular extracts (Figure 1F; Figure S3C). Importantly, PGE_2_ production was also confirmed in ex vivo‐stimulated primary ILC3 (Figure 1G).

### 3.2 PGE_2_ Supports Type 3 Cytokine Production From Human ILC3

PGE_2_ signals through four distinct G‐protein‐coupled receptors (EP1‐4), encoded by *PTGER1*‐*4*. Transcriptomic analysis revealed that *PTGER1* and *PTGER3* were expressed at very low to undetectable levels in ILC3, whereas *PTGER4* (encoding EP4) was highly expressed, alongside lower levels of *PTGER2* (encoding EP2) (Figure 2A; Figure S4A). To investigate the functional impact of PGE_2_ signalling, we stimulated tonsillar ILC3 ex vivo with combinations of cytokines in the presence or absence of exogenous PGE_2_. Consistent with reports on mouse ILC3 [14], PGE_2_ synergized with IL‐2, IL‐1β, and IL‐23 to enhance IL‐17F and IL‐22 expression (Figure 2B–D; Figure S4B). Notably, IL‐17A expression remained unaffected (Figure S4C) and was therefore not included in subsequent ILC3 experiments. Considering that cytokine‐induced PGE_2_ production from ILC3 was low compared with other immune cells, such as alveolar macrophages [38] or monocytes [39], we hypothesized that PGE_2_ may function predominantly in a local auto‐/paracrine manner, mirroring the function of PGD_2_ in ILC2 activation [12]. Supporting this, blockade of PGE_2_ signalling via combined antagonism of EP2 and EP4 during cytokine stimulation significantly reduced IL‐22 and IL‐17F concentrations in culture supernatants (Figure 2E; Figure S4D). Importantly, these experiments were conducted without the addition of exogenous PGE_2_, indicating that ILC3‐derived PGE_2_ contributes to a local feedback loop supporting type 3 cytokine production. PGE_2_ did not support IL1R1 or IL‐23R expression in stimulated ILC3, suggesting that the enhancement of IL‐17F and IL‐22 production is not driven by increased expression of receptors for IL‐1β or IL‐23 (Figure S4E,F). We also observed that PGE_2_ further enhanced COX‐2 expression in ILC3 (Figure S4G,H), suggesting a potential positive feedback mechanism within the PGE_2_ biosynthetic pathway.

**FIGURE 2:**
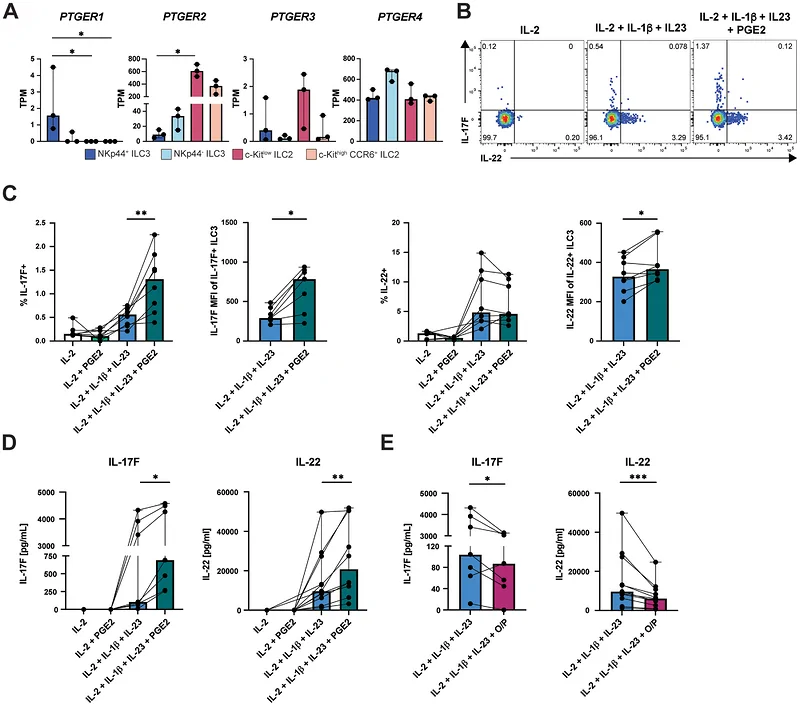
PGE_2_ drives ILC3 effector function, supporting type 3 cytokine production. (A) Expression of PGE_2_ receptors *PTGER1‐4* in human tonsillar ILC3 and blood‐derived ILC2 subsets (*n* = 3 per subset), assessed by RNA‐seq. Data expressed as transcripts per million (TPM); statistical significance was determined using Kruskal–Wallis with Dunn's multiple comparisons test. (B) Flow cytometric analysis of IL‐17F and IL‐22 expression in tonsillar ILC3 stimulated ex vivo for 24 h, with and without exogenous PGE_2_. PMA/ionomycin and Golgi‐Stop/Plug were spiked in for the final 3 h of stimulation. (C) Frequency and expression levels of IL‐17F and IL‐22 in tonsillar ILC3 after 24 h ex vivo stimulation, with and without exogenous PGE_2_ (*n* = 6–8). PMA/ionomycin and Golgi‐Stop/Plug were spiked in for the final 3 h of stimulation. Full cytokine stimulations with and without exogenous PGE_2_ were compared using a Wilcoxon matched‐pairs signed‐rank test. (D) IL‐17F (*n* = 7) and IL‐22 (*n* = 10–11) concentrations in tonsillar ILC3 supernatants, stimulated *ex vivo* for 72 h, as measured by ELISA. Full cytokine stimulations with and without PGE_2_ were compared using a Wilcoxon matched‐pairs signed‐rank test. (E) IL‐17F (*n* = 7) and IL‐22 (*n* = 11) concentrations in tonsillar ILC3, stimulated ex vivo for 72 h with and without EP2 and EP4 antagonists ONO‐AE3‐208 and PF‐04418948 (“O/P”). Both groups were compared using the Wilcoxon matched‐pairs signed‐rank test. (B–E) 10,000 ILC3 were seeded per stimulation condition. All bars show median and range. Significance levels: *p < 0.05, **p < 0.01, ***p < 0.001.

### 3.3 PGE_2_ Production Is Induced During ILC2‐to‐ILC3 Plasticity

Given the distinct prostaglandin biosynthesis and responses between ILC2 and ILC3, we next asked whether these metabolic features are dynamically regulated in the context of ILC plasticity. We sorted c‐kit^low^ (mature ILC2) and c‐Kit^high^ (plastic ILC2) ILC2 from blood and tonsil and exposed them to an ILC3‐polarizing stimulus consisting of IL‐2, IL‐7, IL‐1β, IL‐23, and TGF‐β (Figure 3A). Following polarization, ILC2 acquired key features of an ILC3‐like phenotype, including downregulation of CRTH2, alongside upregulation of RORγt, and induction of type 3 cytokines IL‐17A and IL‐17F (Figure 3B–D; Figure S5A,B). In contrast, IL‐22 expression remained unaffected in both blood and tonsil‐derived polarized cells (Figure 3C; Figure S5A,B).

**FIGURE 3:**
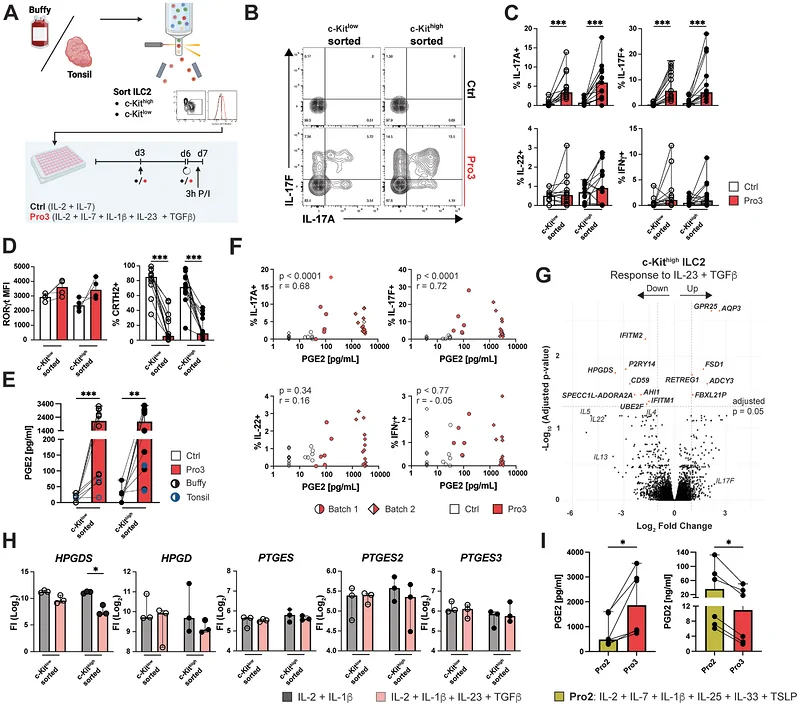
PGE_2_ is induced during ILC2‐to‐ILC3 plasticity. (A) Experimental workflow for ILC2‐to‐ILC3 plasticity stimulation of c‐Kit^high^ and c‐Kit^low^ ILC2 isolated from human tonsil or blood. (B) Flow cytometric analysis of IL‐17A and IL‐17F expression in ILC3‐like cells following ILC3 polarization of blood‐derived ILC2. (C) Expression of type 3 cytokines and IFN‐γ in ILC3‐like cells polarized from blood c‐Kit^high^ and c‐Kit^low^ ILC2 (*n* = 13). (D) Expression of RORγt and CRTH2 in ILC3‐like cells (*n* = 3 for RORγt, *n* = 13 for CRHT2). (E) PGE_2_ concentrations in supernatants of ILC3‐like cells polarized from blood (*n* = 8) and tonsil (*n* = 2) ILC2, as measured by ELISA. (F) Correlation between type 3 cytokines or IFN‐γ, and PGE_2_ levels measured after ILC3 polarization by flow cytometry and ELISA, respectively. Correlation was calculated using Spearman´s test. Spearman r and significance level are indicated. Data from two different ELISA experiments are indicated by circle (“batch 1”) and diamond (“batch 2”). (G) Volcano plot, depicting differential gene expression in c‐Kit^high^ ILC2 following polarization with IL‐2 + IL‐1β + IL‐23 + TGFβ compared with IL‐2 + IL‐1β, assessed by microarray analysis (*n* = 3 donors). Top differentially expressed genes (adjusted *p*‐value < 0.05 and absolute log2 fold change >1) are highlighted in red. Selected, non‐significant genes are additionally indicated. (H) Expression of genes involved in PGD_2_ and PGE_2_ synthesis and degradation in c‐Kit^low^ and c‐Kit^high^ ILC2 stimulated with IL‐2 + IL‐1β or ILC3‐polarizing cocktail IL‐2 + IL‐1β + IL‐23 + TGFβ (*n* = 3). Data are expressed as log2 fluorescence intensity (FI); statistical significance was determined using a moderated t‐test with empirical Bayes trend adjustment, adjusting p‐values for multiple testing using the Benjamini‐Hochberg method. (I) PGE_2_ and PGD_2_ concentrations in bulk ILC2 derived from blood following ILC3‐polarization or alarmin stimulation cultures (“Pro2”: IL‐2 + IL‐7 + IL‐1β + IL‐25 + IL‐33 + TSLP), as measured by ELISA (*n* = 6). (B–F, I) Flow cytometric analysis and ELISA assays were performed after 24 h restimulation (including 3 h of PMA/ionomycin) of 6‐day cultures. (C–E, I) Control or Pro3 and Pro2 conditions were compared using a Wilcoxon matched‐pairs signed‐rank test. All bars show median and range. Significance levels: *p < 0.05, **p < 0.01, ***p < 0.001.

In parallel with these phenotypic changes, ILC3‐like cells exhibited an induction of PGE_2_ production (Figure 3E). PGE_2_ concentrations in supernatants positively correlated with IL‐17A and IL‐17F expression (Figure 3F), suggesting that PGE_2_ biosynthesis accompanies acquisition of a type 3 cytokine program. No correlation was observed between PGE_2_ and IL‐22 or IFN‐γ (Figure 3F).

To explore the mechanistic underpinnings of this metabolic reprogramming, we reanalyzed microarray data of ILC2 cultured under ILC3‐polarizing conditions [31]. While hallmark ILC2 genes, including *IL5* and *IL4*, exhibited a strong downward trend (>twofold reduction), we identified *HPGDS* as one of the top downregulated genes, particularly in c‐kit^high^ ILC2 (Figure 3G,H; Figure S5C), indicating a diminished capacity for PGD_2_ synthesis upon exposure to the type 3‐polarizing stimulus. In contrast, transcript levels of the PGE_2_‐synthases *PTGES1‐3* remained unchanged (Figure 3H). In addition, we observed stable expression levels of *HPGD*, suggesting that the observed PG shift primarily reflects redistribution of prostaglandin synthesis rather than altered degradation (Figure 3H). Corresponding with these transcriptional changes, ILC3‐polarized ILC2 (Figure S5D) exhibited reduced PGD_2_ and increased PGE_2_ production compared with ILC2 stimulated under type 2‐polarizing (“Pro2”) conditions (Figure 3I), consistent with the subset‐specific biosynthetic profiles observed in prototypical ILC subsets.

### 3.4 The Inhibitory Effect of PGE_2_ Is Maintained in Hybrid ILC2/ILC3

Given that PGE_2_ inhibits ILC2 activation [11], while supporting ILC3 effector function, we next asked whether PGE_2_ might promote ILC2‐to‐ILC3 plasticity. Supporting this hypothesis, PGE_2_ has been shown to enhance T_H_17 polarization in human CD4^+^ T cells [40]. To test this, we sorted peripheral blood c‐kit^high^ and c‐kit^low^ ILC2 and subjected them to ILC3‐polarizing conditions, supplementing the Pro3 cytokine cocktail with PGE_2_. Exogenous PGE_2_ did not further reduce the expression of CRTH2, nor did it significantly alter the expression of RORγt or GATA3, and cell viability remained unaffected (Figure 4A). In contrast, PGE_2_ significantly reduced expression of type 3 cytokines IL‐17A, IL‐22, as well as IFN‐γ, while IL‐5 trended downward (Figure 4B). In addition to suppressing cytokine production, exogenous PGE_2_ markedly reduced cellular expansion in ILC2‐to‐ILC3 plasticity cultures (Figure S6A). CellTrace dilution assays confirmed this observation, revealing a reduced proportion of cells undergoing cellular divisions in the presence of PGE_2_ supplementation (Figure 4C; Figure S6B). These findings indicate that the inhibitory PGE_2_ response characteristic of ILC2 is retained during ILC2‐to‐ILC3 plasticity.

**FIGURE 4:**
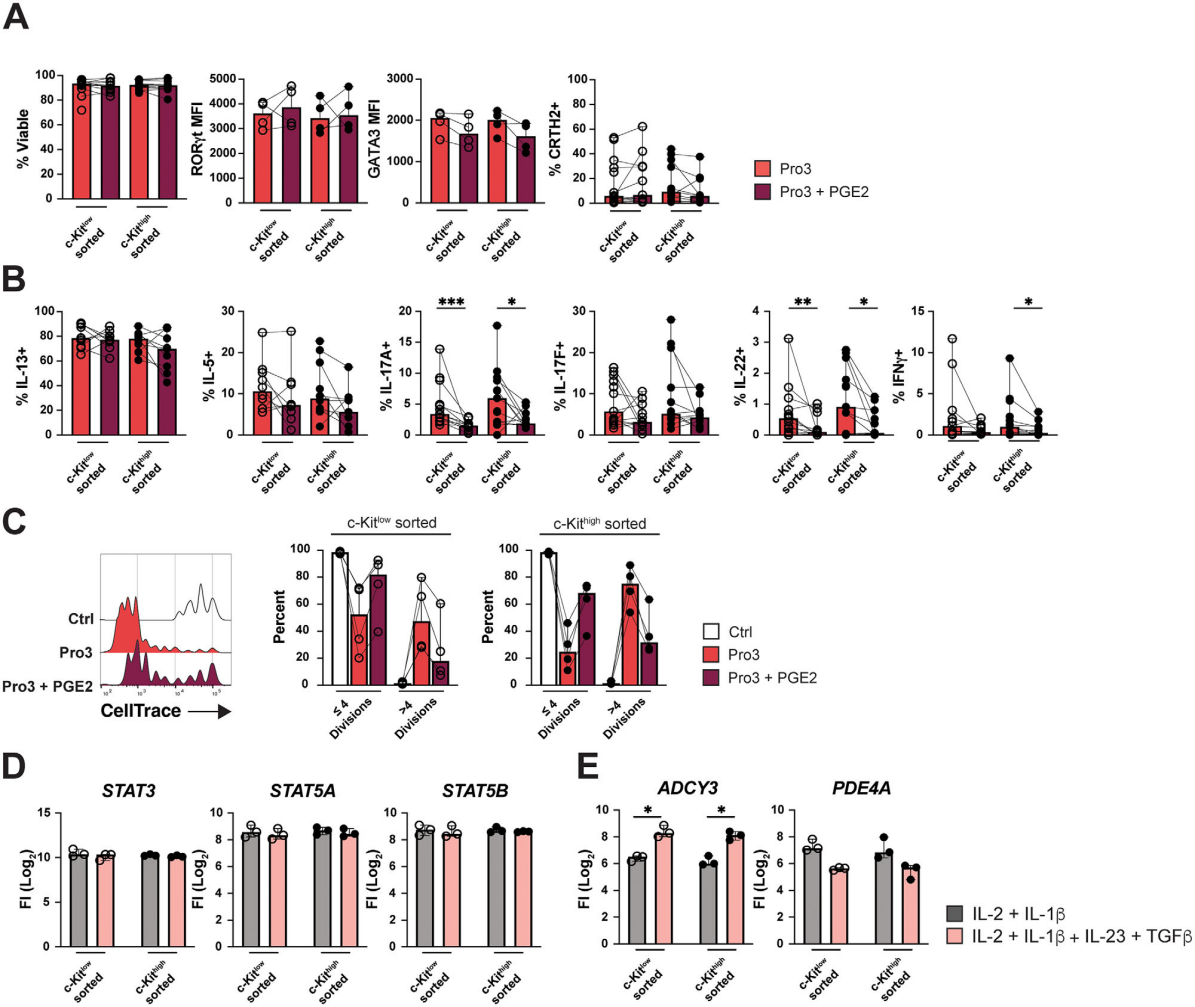
PGE_2_ maintains its inhibitory effect on ILC3‐polarized cells. (A) Viability and expression of hallmark transcription factors RORγt and GATA3, CRTH2, and (B) type 2 and type 3 cytokine production and in ILC3‐polarized c‐Kit^low^ and c‐Kit^high^ ILC2 with and without exogenous PGE_2_ added to the Pro3 polarizing cocktail. (C) Proliferation of polarization cultures with and without exogenously supplemented PGE_2_. Cells were stained with CellTrace at day 0. Stacked histogram showing ILC3‐polarized cells from c‐Kit^high^‐sorted ILC2 of one representative donor. Bar graphs represent the frequency of cells that have undergone ≤ 4 or > 4 divisions over the course of the ILC3‐polarizing culture (*n* = 4). (D) Expression of STAT genes and (E) central enzymes involved in cAMP synthesis and degradation in c‐Kit^low^ and c‐Kit^high^ ILC2 stimulated with IL2 + IL‐1β or ILC3‐polarizing cocktail including IL‐2 + IL‐1β + IL‐23 + TGFβ (*n* = 3), as assessed by microarray analysis. (A, B) Control and Pro3 conditions were compared using a Wilcoxon matched‐pairs signed‐rank test. (A–C) Flow cytometric analysis was performed after 24 h restimulation (including 3 h with PMA/ionomycin) of 6‐day plasticity cultures. (D, E) Microarray data are expressed as log2 fluorescence intensity (FI); statistical significance was determined using a moderated *t*‐test with empirical Bayes trend adjustment, adjusting p‐values for multiple testing using the Benjamini–Hochberg method. All bars show median and range. Significance levels: *p < 0.05, **p < 0.01, ***p < 0.001.

To explore the mechanistic basis for this functional divergence, we examined the expression of STAT5A/B and STAT3, key transcription factors associated with ILC2 [4, 41] and ILC3 [41, 42, 43] function, respectively. While *bona fide* ILC3 were characterized by high *STAT3* levels (Figure S6C), ILC3‐polarization failed to upregulate *STAT3* and did not alter *STAT5A/B* expression (Figure 4D). We further assessed whether ILC2‐to‐ILC3 plasticity affected the cAMP signalling axis, which is canonically induced downstream of EP2 and EP4, and linked to STAT3 phosphorylation and IL‐22 production in mouse ILC3 [14]. *Bona fide* ILC3 expressed high levels of both the adenylyl cyclase *ADCY3* and the phosphodiesterase *PDE4A* (Figure S6D), indicative of a high‐turnover cAMP signalling environment. In contrast, while *ADCY3* was among the most upregulated genes in both c‐Kit^high^ and c‐Kit^low^ ILC2 following ILC3 polarization (Figures 4E and 3G; Figure S5C), *PDE4A* expression showed a trend towards reduced expression (Figure 4E), suggesting an incomplete reprogramming of the cAMP machinery, characterized by an increased capacity for cAMP production but lacking the corresponding cAMP hydrolysis capacity found in mature ILC3.

Taken together, these dynamics demonstrate that ILC2‐to‐ILC3 plasticity does not fully rewire lineage‐intrinsic signalling programs, a finding further supported by hybrid effector states such as the concurrent expression of IL‐17A/F and IL‐13 (Figure S6E). Although ILC3‐polarized cells acquired an increased capacity for cAMP production, the lack of a corresponding shift in other lineage‐specific programs, such as STAT3 expression, provides a framework to explain the persistence of the ancestral ILC2 inhibitory response to PGE_2_.

## 4 Discussion

Lipid mediators are central regulators of immune function and play a well‐established role in shaping ILC2 effector function, with distinct eicosanoid species exerting either activating or inhibitory effects. Moreover, ILC2 produce PGD_2_, which establishes an auto‐/paracrine signalling loop required for their activation. In contrast, much less is known about how lipid mediators regulate ILC3 function.

Here, we demonstrate that prostaglandin metabolism and signalling constitute integral components of both ILC2 and ILC3 effector programs. While both subsets rely on auto‐/paracrine prostaglandin signalling, they exhibit distinct biosynthetic profiles and functional responses. We show that human ILC3 produce PGE_2_ in response to cytokine stimulation, which enhances IL‐17F and IL‐22 production. Moreover, PGE_2_ biosynthesis is induced during ILC2‐to‐ILC3 plasticity and correlates with IL‐17A and IL‐17F expression. In contrast, the inhibitory response to PGE_2_ characteristic of *bona fide* ILC2 is retained, indicating a key difference between ILC2‐derived ILC3‐like cells and *bona fide* ILC3.

PGE_2_ alone did not elicit cytokine expression but synergized with IL‐2, IL‐1β, and IL‐23 to drive IL‐17F and IL‐22 production in ILC3. This aligns with observations in activated Th17 [40, 44] and γδ T cells [45], where PGE_2_ enhances IL‐17 expression when combined with IL‐1β and IL‐23, suggesting a conserved stimulatory role for PGE_2_ across type 3 lymphocytes. PGE_2_ has also been shown to support IL‐22 production in CRTH2^−^CD117^+^ ILC, isolated from human blood [14]. Although originally annotated as ILC3, peripheral CRTH2^−^CD117^+^ ILC largely represent a heterogeneous pool of multi‐ and unipotent ILC precursors (ILCp) [46] rather than ILC3, which are exclusively tissue‐resident. However, ILCp were stimulated with PGE_2_ in combination with IL‐2, IL‐1β, and IL‐23, a cytokine milieu known to drive ILC3 polarization [31, 46, 47], thus providing supporting evidence for a stimulatory role of PGE_2_ in human ILC3, consistent with our data.

PGE_2_ signals through four G protein‐coupled receptors (EP1‐4) that engage distinct intracellular pathways. EP1 and EP3 increase intracellular calcium and decrease cAMP, respectively, whereas EP2 and EP4, expressed on ILC2 and ILC3, activate cAMP‐dependent signaling [48]. In mice, EP4 activation induces the expression of type 3 hallmark genes in intestinal ILC3 [14]. Furthermore, cAMP‐signaling supports type 3 cytokine production in ILC3 [49, 50]. Accordingly, PGE_2_ may enhance ILC3 effector function by providing cAMP‐mediated co‐stimulatory signals that augment IL‐1β‐driven NF‐κB and AP‐1 activation and STAT3 signaling downstream of IL‐23.

Although PGE_2_ was reported to upregulate IL‐23R and IL1R1 expression in CD4^+^ T cell subsets [40], increasing sensitivity to IL‐23 and IL‐1β, we did not observe such receptor induction in ILC3, suggesting that PGE_2_‐dependent modulation of cytokine responsiveness is cell type‐ or context‐dependent.

Our findings broaden the conceptual framework of lipid mediator biology in ILCs, revealing that distinct ILC subsets are differentially wired to produce and depend on specific eicosanoid circuits to shape their effector responses. While ILC3 produce PGE_2_, ILC2 predominantly generate PGD_2_ [12]. Recent studies in mice demonstrated that ILC3 express COX‐2 and mPGES‐1 and produce PGE_2_ upon IL‐1β stimulation, driving *Hbegf* expression via auto‐/paracrine EP2 signaling [15]. Our findings extend these observations to human ILC3, showing that cytokine‐induced PGE_2_ production supports ILC3 effector function via auto‐/paracrine mechanisms. Notably, whereas mouse ILC3 express *Ptges* [15] (encoding mPGES‐1), human ILC3 lack *PTGES* and instead express *PTGES2* and *PTGES3*, highlighting species‐specific differences in PGE_2_ biosynthesis.

Although *PTGES2* and *PTGES3* were expressed in both ILC2 and ILC3, PGE_2_ production was largely restricted to ILC3. However, ILC2 selectively expressed high levels of *HPGDS*, suggesting that the shared precursor PGH_2_ is preferentially channelled into the PGD_2_ biosynthetic pathway, limiting substrate availability for PGE_2_ synthesis. Consistent with this, *HPGDS* was among the top downregulated genes in c‐KIT^high^ ILC2 following type 3 polarization, suggesting a reduced capacity for PGD_2_ synthesis. Correspondingly, ILC3‐polarized ILC2 exhibited reduced PGD_2_ production alongside increased PGE_2_ synthesis, suggesting that prostaglandin biosynthesis is reprogrammed during ILC2‐to‐ILC3 plasticity through metabolic substrate redistribution.

COX‐2 expression is induced during inflammation and is regulated by the coordinated activity of multiple transcription factors in a cell type‐specific manner [51]. In contrast to mouse ILC3, where IL‐1β potently induces COX‐2 expression [15], human ILC3 required combined stimulation with IL‐2, IL‐1β, and IL‐23, suggesting synergistic regulation involving NF‐κB, AP‐1, and STAT signalling pathways. Although PMA/ionomycin was required to induce robust PGE_2_ release, cytokine stimulation alone generated sufficient PGE_2_ to support auto‐/paracrine‐driven enhancement of IL‐17F and IL‐22 production. The further increase in PGE_2_ following ionophore treatment suggests that either human ILC3 intrinsically produce only low levels of PGE_2_, or that IL‐2, IL‐1β, and IL‐23 alone are insufficient to drive maximal PGE_2_ production.

Functionally, blockade of auto‐/paracrine PGE_2_ signalling during ILC3 activation reduced IL‐17F and IL‐22 production by ∼30% and ∼50%, respectively. This contrasts with ILC2, where disruption of the PGD_2_ auto‐/paracrine circuit abolishes effector responses [12]. Together, these results indicate that although PGE_2_ significantly contributes to ILC3 effector function, it may not be as indispensable as PGD_2_ is for ILC2. In tissues, PGE_2_ can also be produced by a plethora of immune and non‐immune cell types that constitute the microenvironment that ILC3 reside in, including monocytes, macrophages, neutrophils, and stromal cells [38, 39, 52]. These additional sources may reinforce, modulate, or override the ILC3‐intrinsic pathway identified here. Local PGE_2_ production and degradation, cellular composition, and spatial organization of these cells may therefore determine the net effect of PGE_2_ on ILC3 within the tissue.

Tissue origin and disease‐associated inflammation may additionally influence prostaglandin responsiveness by altering local availability of lipid mediators, expression of their cognate receptors, and ILC subset composition. Transcriptomic analysis of ILC populations isolated from different human tissues revealed broad but heterogeneous expression of EP2 and EP4, and CRTH2 among ILC populations from blood, lung, tonsil, and colon [29]. While receptor transcript abundance does not directly predict surface availability and functional signalling, these findings suggest the possibility of tissue‐specific differences in responsiveness to prostaglandins such as PGE_2_ and PGD_2_. Moreover, inflammatory disease may alter the relative abundance of ILC2, ILC3, and induce hybrid phenotypes with distinct intrinsic prostaglandin responses. Direct analysis of ILCs from disease‐relevant tissues will therefore be required to determine how the PGE_2_ and PGD_2_ circuits, including their potential opposing effects identified here, operate in the context of pathology.

ILC2 exhibit pronounced plasticity and hybrid ILC2/ILC3 phenotypes, expressing both type 2 and type 3 markers, and have been described in diseases such as mixed granulocytic asthma [33], atopic dermatitis [53], and cystic fibrosis [32], suggesting that ILC plasticity may contribute to disease by enabling the emergence of hybrid effector states.

Our observation that ILC2 induce PGE_2_ biosynthesis upon ILC3 polarization extends this plasticity into the lipid mediator domain. PGE_2_ has previously been shown to support Th17 polarization in naïve CD4^+^ T cells, when combined with IL‐1β and IL‐23 [40], consistent with a role in promoting type 3‐associated programs. However, despite adopting ILC3‐like cytokine production and prostaglandin biosynthesis, ILC3‐polarized ILC2 retained the inhibitory response to PGE_2_ characteristic of their ILC2 origin. These findings suggest that although ILC2 exhibit phenotypic plasticity, hybrid ILC2/ILC3 cells are distinct from *bona fide* ILC3, at least with respect to lipid mediator signalling. While ILC2‐to‐ILC3 plasticity enhanced expression of cAMP‐associated signalling components important for ILC3 function [49, 50], STAT expression itself remained unchanged, suggesting preserved lineage‐intrinsic signalling constraints that may explain sustained sensitivity to PGE_2_‐mediated inhibition. Further investigation of the signalling machinery, including STAT utilization and cAMP signalling, will be needed to determine which signalling features distinguish hybrid ILC2/3 from *bona fide* ILC3 and underlie their differential responses to PGE_2_.

In summary, our findings uncover a previously unrecognized layer of ILC3 regulation in which PGE_2_ functions as an intrinsic auto‐/paracrine regulator of effector function and establish auto‐/paracrine eicosanoid signalling as a shared feature of ILC biology extending beyond ILC2. The persistence of PGE_2_‐mediated inhibition in ILC3‐polarized ILC2 reveals a functional constraint of ILC plasticity, whereby phenotypic and metabolic adaptation is uncoupled from downstream signalling responsiveness. These insights may have implications for inflammatory conditions characterized by mixed ILC2/ILC3 phenotypes, where targeted modulation of prostaglandin pathways could suppress inflammation even in phenotypically altered ILC populations.

## Author Contributions

L.W. designed the study, performed and analyzed experiments, and wrote the manuscript. W.W. and E.K. supported the experimental work. J.K., A.‐K.J., A.Q., and C.W. performed LC‐MS analyses, processed mass‐spectrometry data, and contributed to MS data interpretation. M.J. provided resources. R.V.P. processed microarray and RNA‐sequencing data. S.E.D., T.H., and C.A.T. contributed to study design, provided conceptual input, and supported data interpretation. J.M. conceptualized the study, supervised, and supported all aspects of this project. All authors contributed to manuscript revision and approved the final version.

## Funding

This project has received funding from the European Union's Horizon 2020 research and innovation programme under the Marie Skłodowska‐Curie grant agreement no. 813343. The study was further supported by funds from the Swedish Heart‐Lung Foundation (to J.M., C.E.W., and S.‐E.D.), the Center for Innovative Medicine (CIMED, to J.M.), the Swedish Research Council (to J.M.), the Swedish state under the agreement between the Swedish government and the county councils, the ALF‐agreement (to J.M., S.‐E.D.), the Torsten Söderberg Foundation (to S.‐E.D.), and the Karolinska Institutet (to J.M. and S.‐E.D.).

## Ethics Statement

This study involves material derived from human samples. All experiments were conducted in accordance with the relevant ethical guidelines, with approval from the Swedish Ethical Review Authority.

## Conflicts of Interest

Lorenz Wirth is a former, and Thomas Hochdörfer is a current AstraZeneca employee. Jenny Mjösberg is an AstraZeneca grant holder for the submitted work and has received honoraria for lectures from AstraZeneca, Chiesi, Novartis and Sanofi outside the submitted work. Craig Wheelock reports institutional grants from AstraZeneca and Cayman Chemicals outside the scope of the current work. Sven‐Erik Dahlén reports institutional grants from AstraZeneca, Cayman Chemicals, GSK and Sanofi and personal honoraria for lectures or advisory boards from Affibody, AstraZeneca, GSK, Sanofi, and Teva. The remaining authors declare no conflicts of interest.

## Supporting information




**Supporting File**: eji70296‐sup‐0001‐SuppMat.pdf.

## Data Availability

Data supporting the findings of this study are available from the authors upon reasonable request.
