# Supplementary material for: PGE2 Enhances Human ILC3 Function but Constrains ILC2‐to‐ILC3 Plasticity
Source: Eur J Immunol. 2026 Oct 8;56(10):e70296. doi: 10.1002/eji.70296 (PMC13647482; doi:10.1002/eji.70296)
Supplement: Supplementary file 1 [file EJI-56-e70296-s001.pdf]

PGE_2 Enhances Human ILC3 Function But Constrains ILC2-to-ILC3 Plasticity

Supplementary Material

- Supplementary Figures 1 – 6
- Supplementary Tables 1-2

Authors

Lorenz Wirth[1,2,3], Whitney Weigel[1,3], Efthymia Kokkinou[4], Johan Kolmert[5], Anna-Karin Johnsson[6], Alessandro Quaranta[5], Craig Wheelock[5,7], Mattias Jangard[8], Ram V. Pandey[9], Sven-Erik Dahlén[3,5,7], Christopher Andrew Tibbitt[1,3], Thomas Hochdörfer[2], Jenny Mjösberg[1,3,#]

[#] Corresponding author

Affiliations

1 Center for Infectious Medicine, Department of Medicine Huddinge, Karolinska Institutet and Karolinska University Hospital, Stockholm, Sweden

2 Mechanistic Biology & Profiling, Discovery Sciences, R&D, AstraZeneca, Gothenburg, Sweden

3 Clinical Lung- and Allergy Research Unit, Department of Medicine Huddinge, Karolinska Institutet, Stockholm, Sweden

4 Bloomberg Kimmel Institute of Cancer Immunotherapy, Department of Oncology, Johns Hopkins University School of Medicine, Baltimore, USA

5 Unit of Integrative Metabolomics, Institute of Environmental Medicine, Karolinska Institutet, Stockholm, Sweden

6 Division of Immunology and Allergy, Department of Medicine Solna, and Center for Molecular Medicine, Karolinska Institutet, and Karolinska University Hospital, Stockholm, Sweden

7 Department of Respiratory Medicine and Allergy, Karolinska University Hospital, Stockholm, Sweden

8 ENT-Unit, Sophiahemmet University and Sophiahemmet Hospital, Stockholm, Sweden

9 Medical University of Vienna, Department of Dermatology, Vienna, Austria.

Corresponding author: Jenny Mjösberg, PhD, Center for Infectious Medicine, Department of Medicine Huddinge, Karolinska Institutet and Karolinska University Hospital, Stockholm, Sweden. E-mail: jenny.mjosberg@ki.se

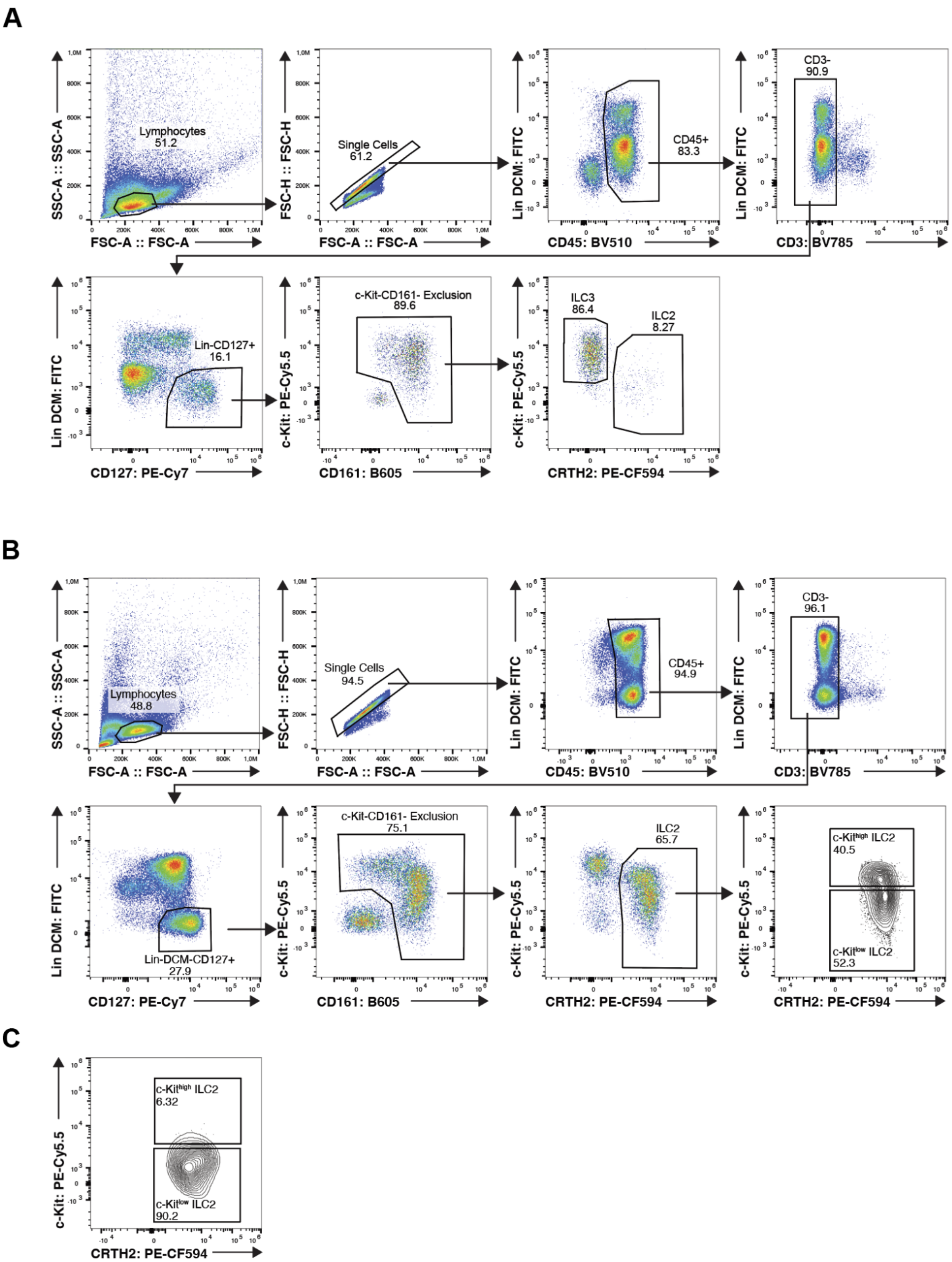

Fig. S1: Gating strategy for isolation of ILC2 from blood and tonsil. (**A**) Gating strategy for FACS-sorting ILC3 and ILC2 from human tonsil following MACS-depletion of $CD3^+CD14^+CD19^+$ mononuclear tonsillar cells. (**B**) Gating strategy for FACS-sorting c-Kit^{high} and c-Kit^{low} ILC2 from peripheral blood, following depletion of $CD3^+$ and

subsequent enrichment CD127^{+} of PBMCs, and (**C**) final gate for FACS-sorting c-Kit^{high} and c-Kit^{low} ILC2 from tonsil following CD3^{+}CD14^{+}CD19^{+} depletion of tonsillar mononuclear cells.

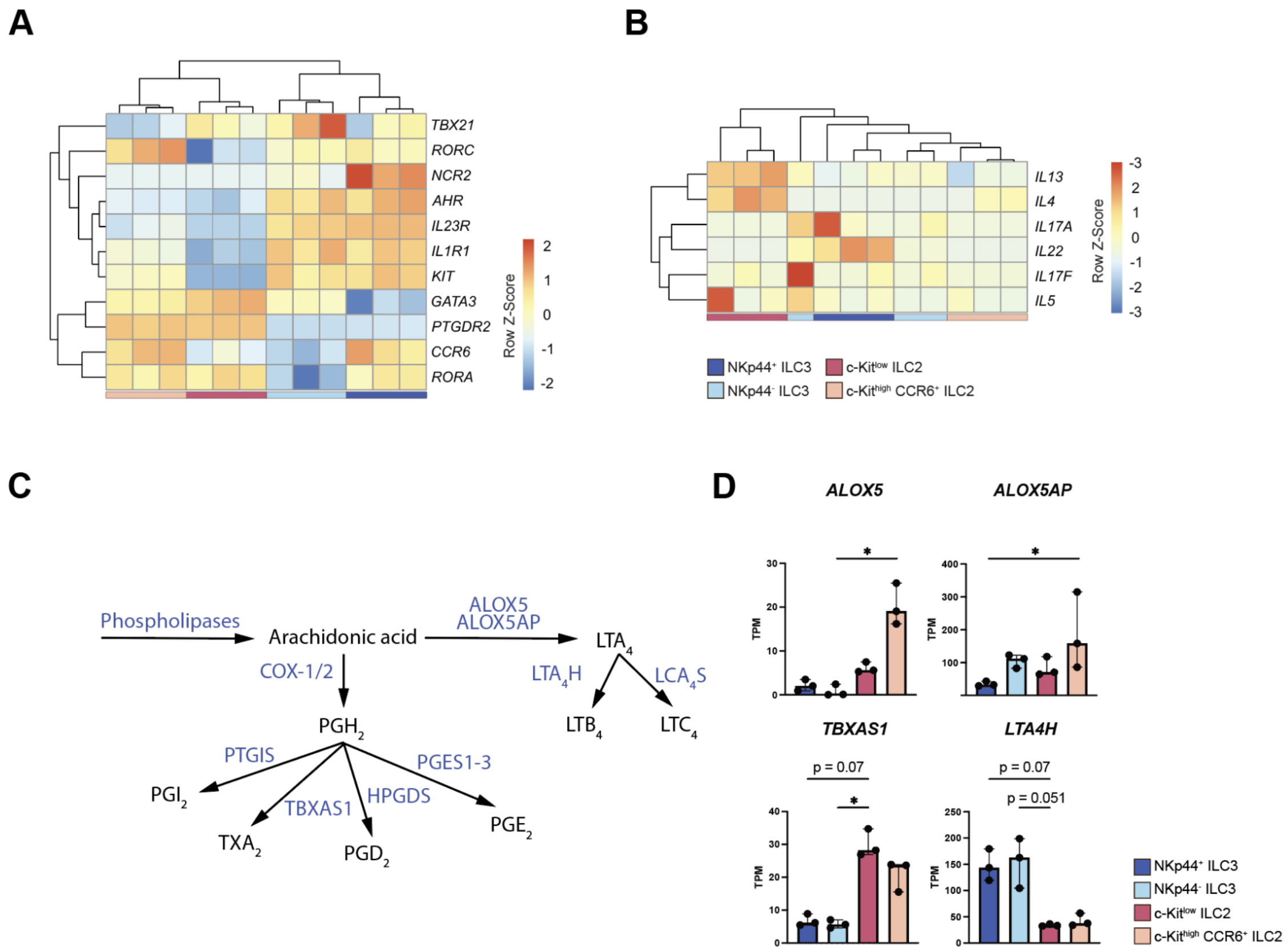

Fig. S2: Characterization of tonsillar ILC subsets and oxylipin synthesis. Heatmap showing relative gene expression of (**A**) hallmark transcription factors and surface markers, and (**B**) cytokines in tonsillar NKp44$^{+/-}$ ILC3 and blood-derived c-Kit$^{low/high}$ ILC2 (*n* = 3 per subset). Color scales represent row Z-scores of TPM values. (**C**) Schematic representation of arachidonic acid metabolism pathway, including prostaglandin and leukotriene synthesis. (**D**) Expression of rate-limiting enzymes involved in prostaglandin and leukotriene synthesis in NKp44$^{+/-}$ ILC3 and c-Kit$^{low/high}$ ILC2. Data are expressed as Transcripts Per Million (TPM); statistical significance was determined using Kruskal-Wallis with Dunn´s multiple comparisons. Bars show median and range. Significance levels: *$p < 0.05$.

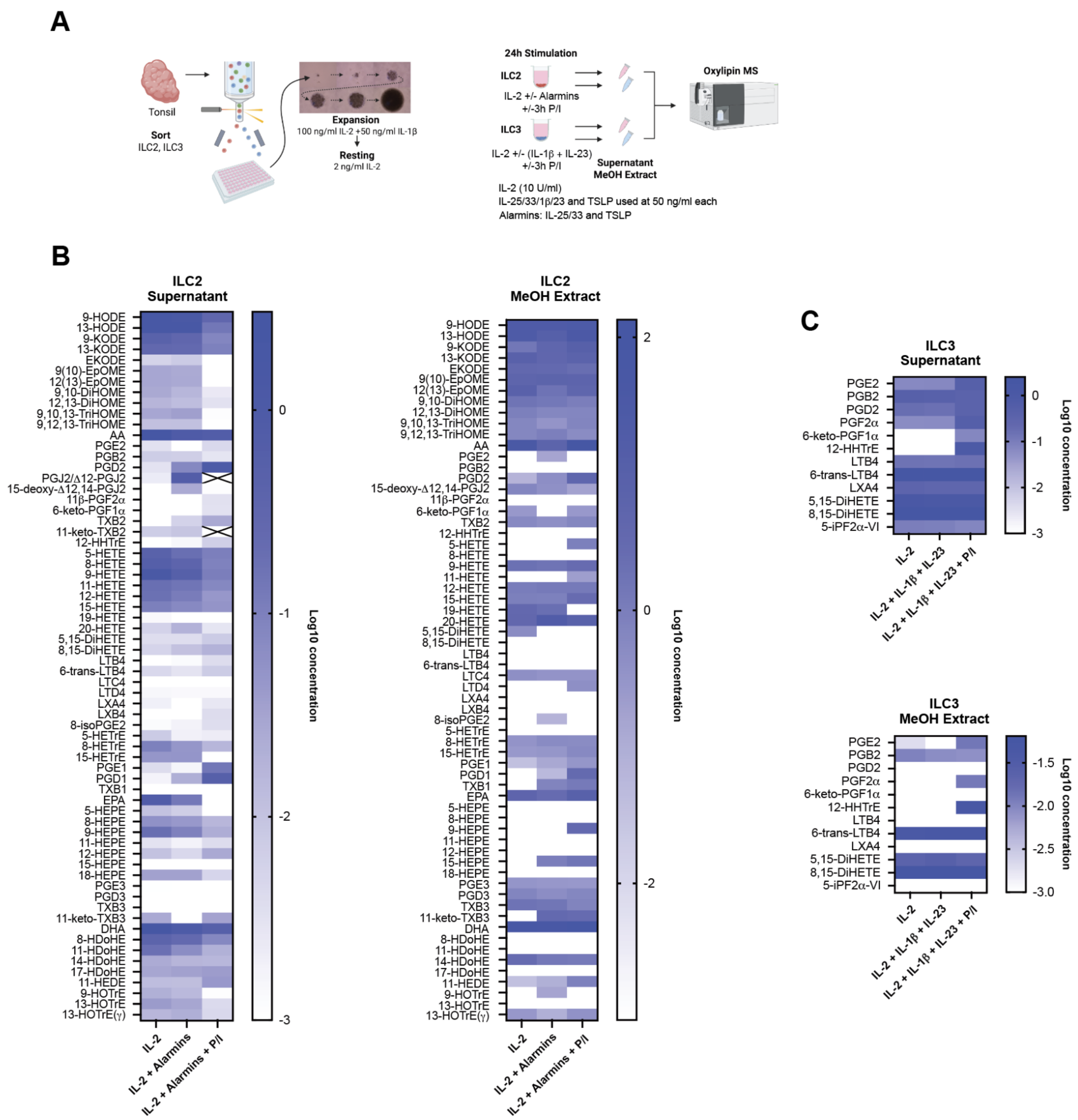

Fig. S3: Oxylipin profiles in ILC2 and ILC3 culture supernatants and cellular extracts. (**A**) Experimental workflow for the isolation, *in vitro* expansion, stimulation, and analysis of oxylipin synthesis in ILC3 and ILC2 using LC-MS/MS. (**B**) Heatmaps showing oxylipins measured and detected in culture supernatants and corresponding cellular extracts of expanded and rested ILC2 following 24 h stimulation under the indicated conditions. The ILC2 heatmaps combine data from two independent LC-MS/MS experiments. (**C**) Heatmaps showing oxylipins measured and detected in culture supernatants and corresponding cellular extracts of expanded and rested ILC3 following 24 h stimulation under the indicated conditions. (**B-C**) Each heatmap cell represents the median normalized concentration for the indicated analyte and condition. Measured concentrations were normalized to an input of 2.5 x 10^5 cells.

Median normalized concentrations were transformed as $\log_{10}(Y+0.001)$ to facilitate visualization of both low- and high-abundance analytes, where Y represents the normalized concentration in ng/mL. 0.001 was added as a pseudocount to accommodate zero values. Crossed cells indicate analyte-condition combinations for which no measurement was performed.

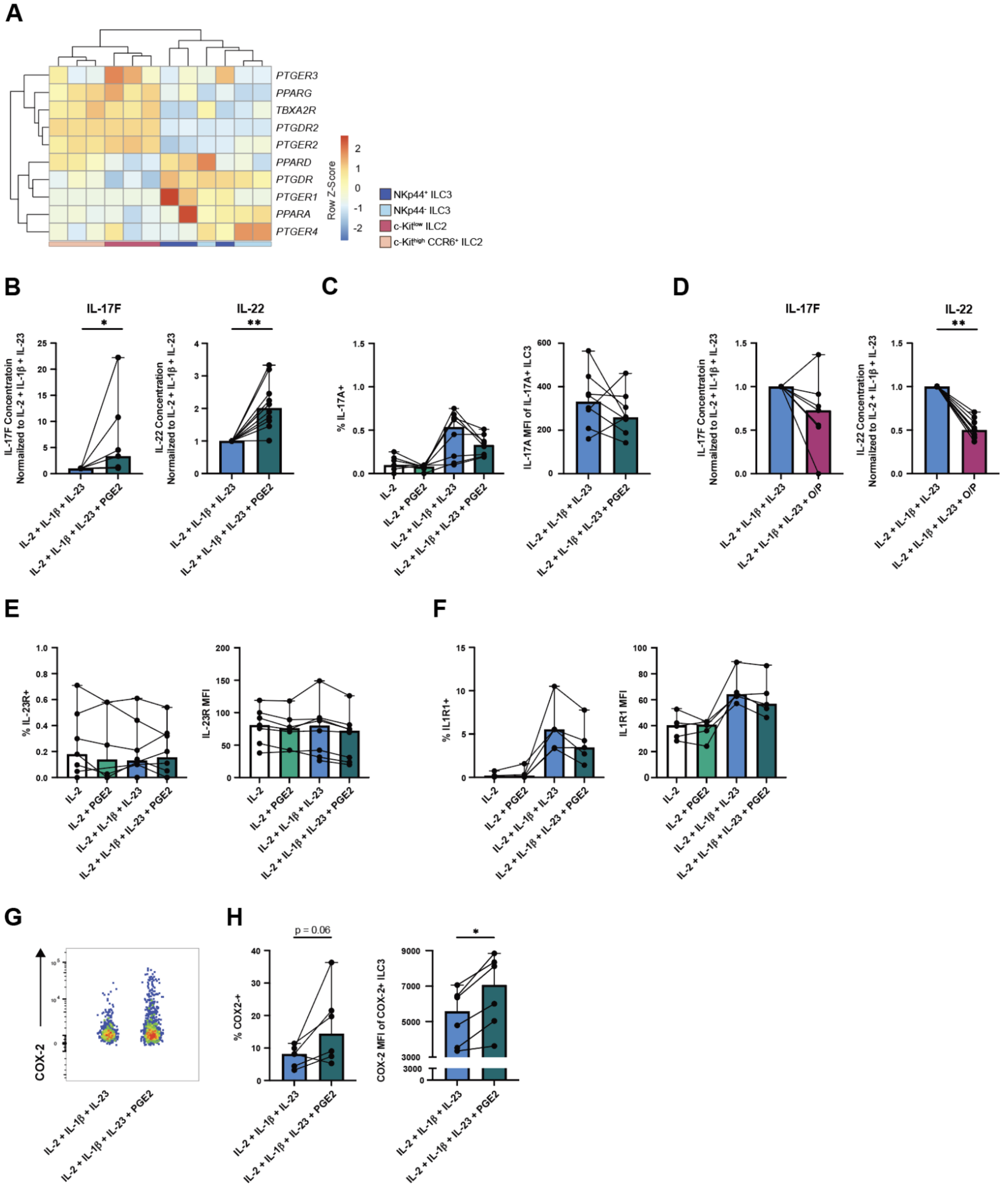

Fig. S4: ILC3 responsiveness to PGE_2 and COX-2 expression. (**A**) Heatmap showing expression of oxylipin receptor genes in tonsillar NKp44$^{+/-}$ ILC3 and blood-derived c-Kit$^{low/high}$ ILC2 (*n* = 3 per subset). Color scale represents row Z-scores of TPM values. (**B**) Fold change of IL-17F (*n* = 7) and IL-22 (*n* = 10) concentrations in ILC3 supernatants stimulated *ex vivo* for 72 h. Concentrations were normalized to cytokine stimulation alone. (**C**) Flow cytometric analysis of IL-17A expression in ILC3 stimulated *ex vivo* for 24 h, with and without exogenous PGE_2 (*n* = 7 – 8).

PMA/ionomycin and Golgi-Stop/Plug were spiked in for the final 3 h of stimulation. (**D**) Fold change of IL-17F (*n* = 7) and IL-22 (*n* = 11) concentrations in ILC3, stimulated *ex vivo* for 72 h with and without EP2 and EP4 antagonists ONO-AE3-208 and PF-04418948 ("O/P"). Concentrations were normalized to cytokine stimulation without antagonists. (**E**) Frequency and MFI of IL-23R and (**F**) IL1R1 in ILC3 stimulated *ex vivo* for 24 h, with and without exogenous PGE_2 (*n* = 4 – 7). (**G**) Flow cytometric analysis of COX-2 expression in ILC3 stimulated 24 h *ex vivo* with cytokines and with or without exogenous PGE_2. (**H**) Frequency and COX-2 expression levels of COX-2^+ ILC3 after 24 h of *ex vivo* stimulation (*n* = 6). (**B**-**F**) 10,000 ILC3 were seeded per stimulation condition. (**B**-**D**, **F**) Statistical difference between groups was assessed using Wilcoxon matched-pairs signed rank test. All bars show median and range. Significance levels: *$p < 0.05$, **$p < 0.01$.

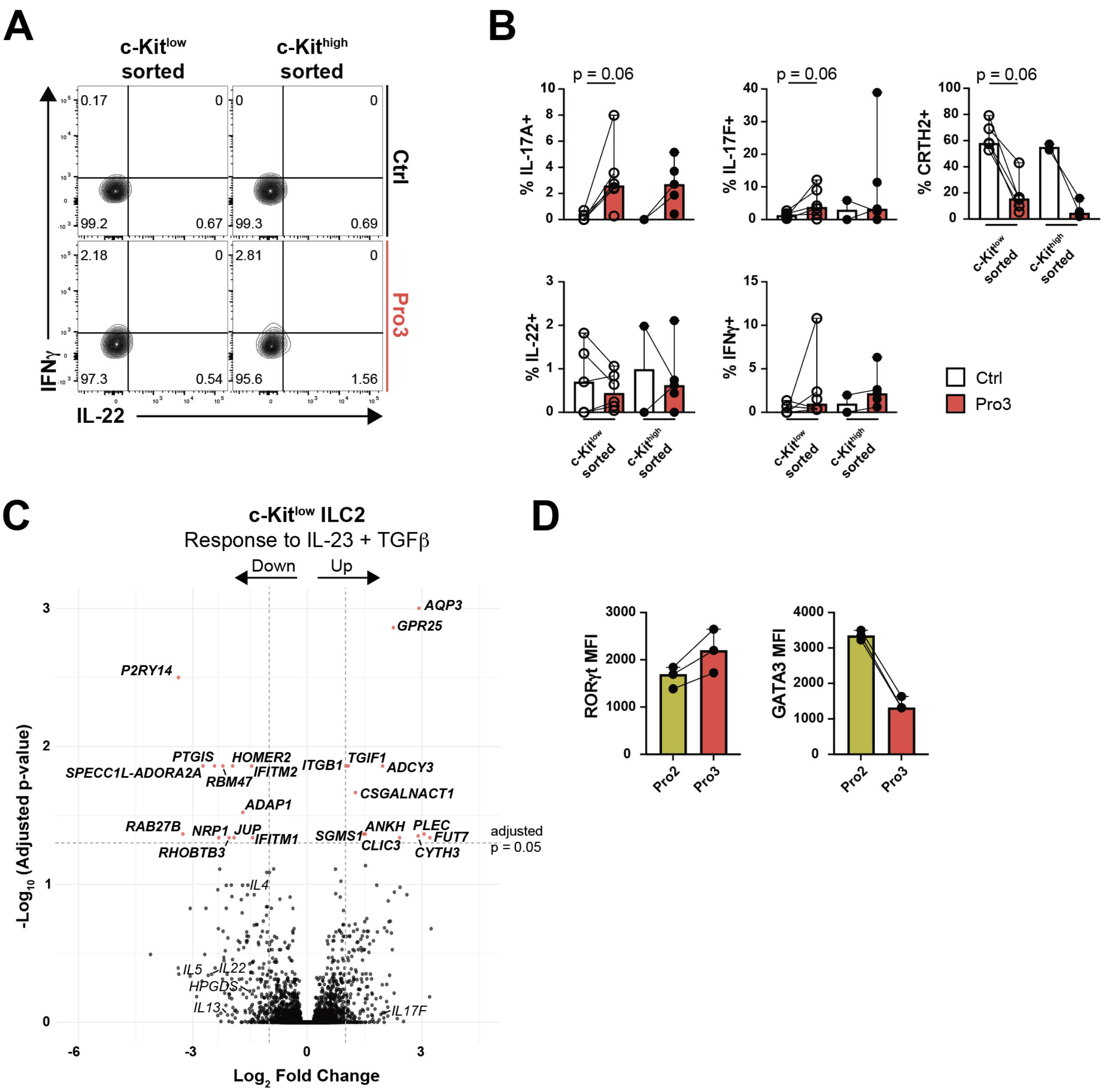

Fig. S5: Extended characterization of ILC2 responses to ILC3-polarizing conditions across tissue and molecular readouts. (**A**) Flow cytometric analysis of of IL-22 and IFN-γ expression in ILC3-like cells following ILC3-polarization of blood-derived ILC2. (**B**) Expression of hallmark type 3 cytokines and CRTH2 after ILC3-polarization of c-Kit^{high} and c-Kit^{low} ILC2 isolated from tonsil (n = 2 – 5). Control and Pro3 conditions were compared using Wilcoxon matched-pairs signed rank test. (**C**) Volcano plot, depicting differential gene expression in blood-derived c-Kit^{low} ILC2 following polarization with IL-2 + IL-1β + IL-23 + TGFβ compared to IL-2 + IL-1β, assessed by microarray analysis (n = 3 donors), serving as the counterpart to the c-Kit^{high} analysis in **Fig. 3G**. Top differentially expressed genes (adjusted p-value < 0.05 and absolute log2 fold change > 1) are highlighted in red. Selected, non-significant genes additionally indicated. (**D**) RORγt and GATA3 MFIs in bulk ILC2 derived from

blood following ILC3-polarization or alarmin stimulation cultures ("Pro2": IL-2 + IL-7 + IL-1β + IL-25 + IL-33 + TSLP), as measured by flow cytometry (n = 3). (**A-B, D**) Flow cytometric analysis was performed after 24 h restimulation (including 3 h of PMA/ionomycin) of 6-day cultures. All bars show median and range.

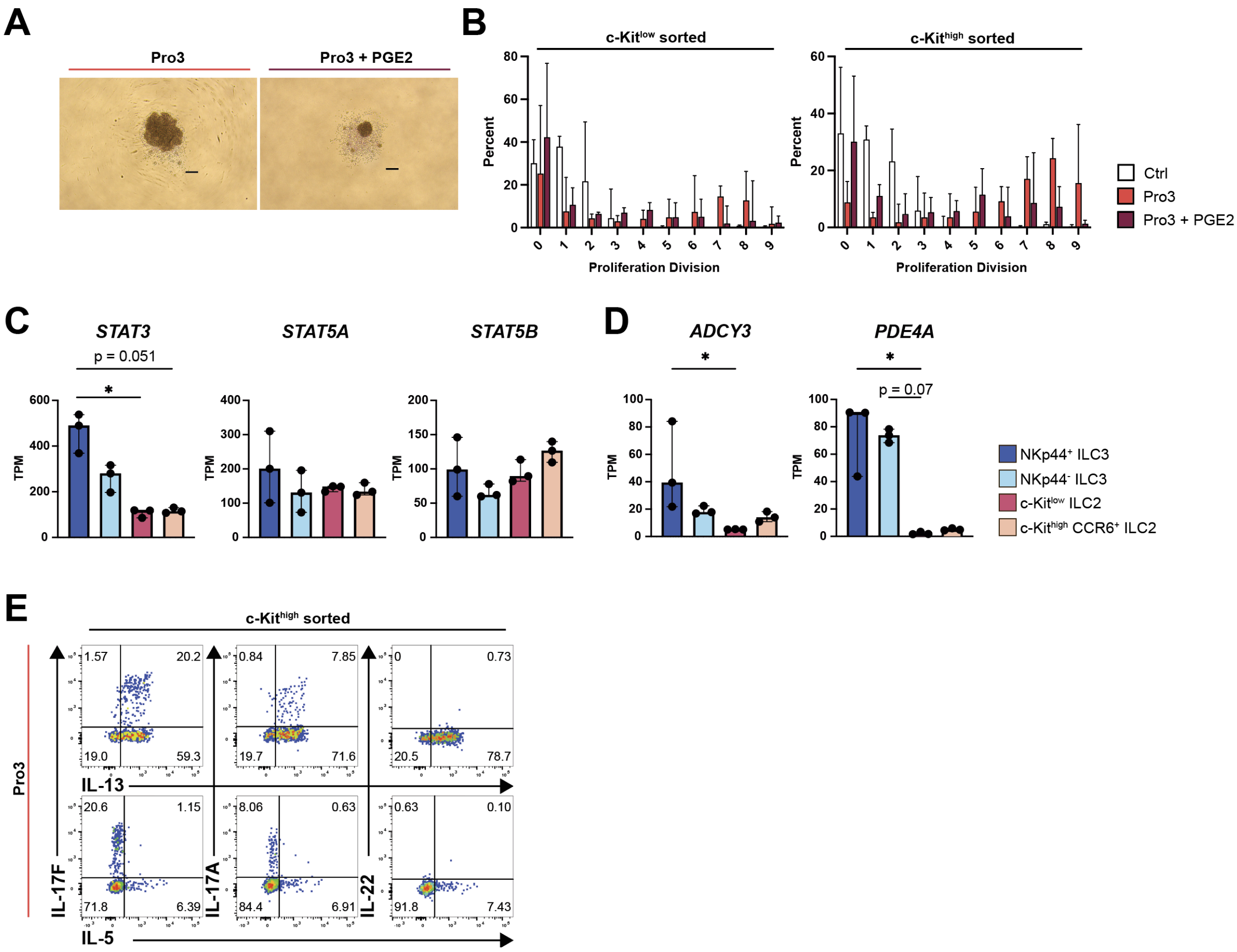

Fig. S6: Morphological and molecular characterization of ILC2-to-ILC3 plasticity. (**A**) Representative brightfield microscopy images of blood derived c-Kit^{high} ILC2 after 6 days of Pro3 polarization with and without exogenous PGE_2 supplementation. 40x magnification; scale bar = 250 μm. (**B**) Proliferation of polarization cultures with and without exogenously supplemented PGE_2. Cells were stained with CellTrace at day 0. Bar graphs represent the frequency of each division relative to the total population (n = 4). (**C**) Expression of *STAT3* and *STAT5A/B* and (**D**) *ADCY3* and *PDE4A* in tonsillar NKp44$^{+/-}$ ILC3 and blood-derived c-Kit$^{low/high}$ ILC2 (n = 3 per subset). RNA-seq data expressed as Transcripts Per Million (TPM); statistical significance was determined using Kruskal-Wallis with Dunn´s multiple comparisons test. (**E**) Flow

cytometric analysis showing co-expression of type 2 and type 3 hallmark cytokines, in ILC3-polarized cells derived from blood-sorted c-Kit^{high} ILC2 in one representative donor. (**B**, **E**) Analysis was performed after 24 h restimulation (including 3 h with PMA/ionomycin) of 6-day plasticity cultures. All bars show median and range. Significance levels: *$p < 0.05$.

Table S1: List of antibodies used for FACS sorting of ILC2 and ILC3.

Target	Fluorochrome	Clone	Vendor	Titration
CD3	BV785	OCT3	Biolegend	2:50
CD161	BV605	HP-3G10	Biolegend	4:50
CD45	V500	HI30	BD	1:50
CD127	PE-Cy7	R34.34	Beckman Coulter	3:50
CD117	PE-Cy5.5	B96754	Beckman Coulter	3:50
CRTH2	PE-CF594	BM16	BD	3:50
Live/Dead Fixable Cell Stain Kit	Green	n.a.	Thermo Fisher	1:2500
CD1a	FITC	HI149	Biolegend	2:50
CD14	FITC	Tuk4	LifeTech	2:50
CD19	FITC	4G7	BD	2:50
CD34	FITC	581	Biolegend	2:50
BDCA2	FITC	AC144	Miltenyi	2:50
CD94	FITC	DX22	Biolegend	2:50
CD123	FITC	6H6	Biolegend	2:50
TCRαβ	FITC	IP26	Biolegend	2:50
TCRγδ	FITC	B1	Biolegend	2:50
FcεR1α	FITC	AER-27	Biolegend	2:50

Table S2: List of antibodies used for flow cytometry analysis of ILC2 and ILC3.

Target	Fluorochrome	Clone	Vendor	Titration
Live/Dead Fixable Cell Stain Kit	Green	n.a.	Thermo Fisher	1:2500
CD127	PE-Cy7	R34.34	Beckman Coulter	3:50
CD117	PE-Cy5.5	B96754	Beckman Coulter	3:50
CRTH2	PE-CF594	BM16	BD	3:50
IL-1R1	AF700	FAB269N	R&D Systems	1:10
IL-23R	PE	FAB14001	R&D Systems	1:50
IL-5	PE	TRFK5	Biolegend	1:50
IL-13	APC	JES-10-5A2	Biolegend	1:50
IL-17A	APC-Cy7	BL168	Biolegend	1:50
IL-17F	BV650	O33-782	BD	1:50
IL-22	PE-Cy7	2G12A41	Biolegend	1:50
IFN-γ	V500	B27	BD	1:50
COX-2	PE	COX229	Invitrogen	1:1000
RORγt	PE	AFKJS-9	Invitrogen	1:50
GATA3	BV711	L50-823	BD	1:50
